# A dereverberation beamforming algorithm for noise source localization in anechoic and semi-reverberant environments

**DOI:** 10.1016/j.mex.2025.103269

**Published:** 2025-03-14

**Authors:** R. Singh, A. Mimani, R. Kumar

**Affiliations:** Department of Mechanical Engineering, Indian Institute of Technology Kanpur, Kanpur 208016, India

**Keywords:** Cross-spectral beamforming, Cross-correlation matrix, Dereverberation, Dereverberation beamforming based on cross-correlation windowing

## Abstract

This paper presents a dereverberation beamforming (DBF) technique based on windowing the cross-correlation matrix (CCM) to improve the localization accuracy of beamforming maps for imaging the noise sources generated by real-world applications. Following a trial-and-error procedure, an optimal frequency-dependent half-window width was determined to retain the direct field by multiplying the CCM with optimized Hanning window. A small loudspeaker and a portable mixer-grinder appliance placed in anechoic and semi-reverberant room environments were considered, and conventional beamforming (CBF) was first implemented. In the anechoic chamber, the CBF could accurately localize the loudspeaker, while for the mixer-grinder, a noticeable localization error was observed, especially at higher frequencies, which is attributed to self-scattering. In the room, the CBF delivered a small localization error for the loudspeaker, while for the mixer-grinder, it performed poorly, rendering it impossible to interpret the maps due to large side-lobes, particularly at high frequencies. By considering only the first few points at low frequencies and increasingly more points at higher frequencies in the CCM window, the DBF accurately localized the sources in both anechoic and semi-reverberant environments within λ/20 uncertainty. Additionally, the CLEAN-SC deconvolution applied to the DBF maps of the mixer-grinder significantly improved the source resolution.

• DBF algorithm is presented based on CCM filtering using an optimized Hanning window to significantly improve the localization accuracy of noise sources within λ/20 uncertainty.
• Trial-and-error method is used to determine the optimal Hanning window width, which depends on frequency, noise source and surroundings.
• It was necessary to implement DBF even in an anechoic chamber to accurately localize noise sources generated by engineering applications.

## Graphical abstract

**Figure:**
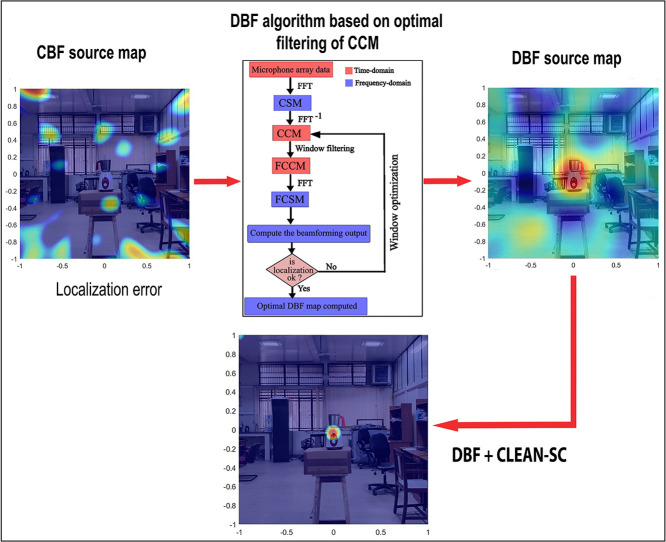

Specifications table

**Table:** 

Subject area:	Engineering
More specific subject area:	Mechanical Engineering, Acoustic array signal processing
Name of your method:	Dereverberation beamforming based on cross-correlation windowing
Name and reference of original method:	• J. Fischer, C. J. Doolan, Improving acoustic beamforming maps in a reverberant environment by modifying the cross-correlation matrix, *J. Sound Vib*. 411 (2017) 129–147. • R. Singh, A. Mimani, Dereverberation and background noise reduction techniques for improving beamforming maps of engineering noise sources, *In Proceedings of the Berlin Beamforming Conference* held on the 9th and 10th June 2022 in *Berlin, Germany*.
Resource availability:	The microphone array data of the noise emitted by the mixer-grinder home appliance can be made available in the form of **.tdms** files on request.

## Background

An acoustic camera based on conventional beamforming (CBF) is now a standard tool for imaging noise sources from engineering applications, which include wind-turbines [1], gas pipeline valve [2], vehicle pass-by noise [3], aerospace structures [4,5], and high-speed trains [6] as well as diagnosing faults in rotating machines [7] and axle-box bearings [8] and identifying grinding mill dynamics [9]. The hardware components include a microphone array located at a certain distance from the source, a data acquisition system (DAQ), and a camera. The recorded data is used to obtain the cross-spectral matrix (CSM) that is used to compute an acoustic image showing the hot spots representing the dominant sources, which is then overlaid on the camera image, wherein one can readily observe the location of the noise source(s) in relation to the application [10]. An important limitation of CBF is that it assumes a free-field propagation, i.e., free-space Green's function of a monopole to compute the steering vectors [[11], [12], [13]]. This approach is less suited in reverberant or semi-reverberant environments, characterized by multiple reflections and several scattering surfaces. In such cases, the CBF often results in large side-lobes that completely mask the main lobe, causing ambiguity in identifying the source [[14], [15], [16]]. Previous investigations attempting to overcome the problems caused by reverberation based on modifying the steering vectors include the method of images [14,17,18] or equivalently, the image source model (ISM) [19]. The underlying idea of these geometry-specific methods is to model reflections due to hard-wall wind tunnel boundaries by placing imaginary image sources and considering the actual source location relative to the physical boundaries. Furthermore, modified steering vectors were also obtained by measuring the experimental Green's function by moving a source at different grid points in a reverberant wind-tunnel test-section, and the corresponding source maps were better than the ISM [14].

More recently, a generic technique has been proposed, which attempts to remove reflections from the data by windowing the cross-correlation matrix (CCM), thereby retaining only the direct field [15,16]. The reflections were filtered out by multiplying CCM time-vectors by an optimized Hanning window centered at the primary peak so that CCM is truncated well before the occurrence of the first reflection peak. The filtered-CCM (FCCM) was converted back to the frequency-domain to deliver the filtered-CSM (FCSM) that was used to compute the beamforming map. This technique, called dereverberation beamforming (DBF), was not geometry-specific and significantly improved the localization for a loudspeaker and an airfoil in a reverberant wind tunnel. However, its limiting feature was that an average optimal half-width of the Hanning window was considered across low-to-high frequencies, which is likely to produce an error and large side-lobes for complex real-world applications for which the optimal half-width is likely to be completely different due to its unique directivity. Here, the objective is to demonstrate the necessity and effectiveness of the improved frequency-dependent CCM windowing-based DBF algorithm to localize sources generated from representative engineering applications such as a commonly used mixer-grinder home-appliance placed in anechoic chamber and a semi-reverberant room environment having a significantly larger reverberation time (RT60), see Refs. [[20], [21], [22]] that makes it even more challenging for source localization.

## Method details

### Dereverberation beamforming (DBF) algorithm

**Fig. 1:**
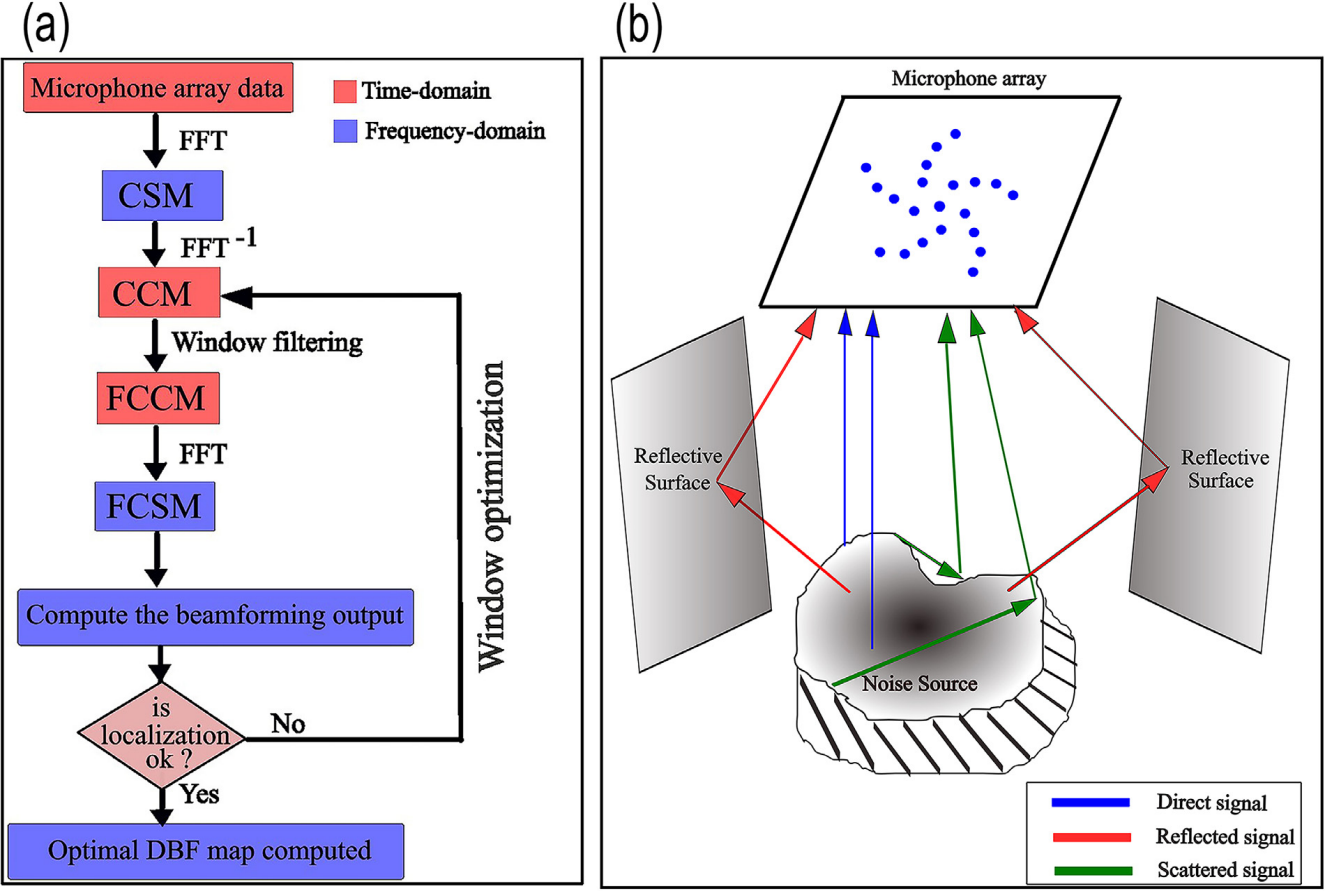
Part (a) A flow-chart summarizing the DBF algorithm based on optimal filtering of the CCM signals for removing reflections. Part (b) Schematic showing acoustic radiation by a noise-producing application in a semi-reverberant environment – the direct, reflected, and self-scattered signals intercepted by a planar microphone array have been annotated.

A generic dereverberation beamforming (DBF) algorithm is presented here, based on filtering out the reflected signals and retaining only the direct acoustic field data that delivers the most accurate noise source localisation whilst minimizing the side-lobes in the source map. The underlying idea is to first obtain the CCM from the CSM computed from the data recorded at the microphone array. Next, a Hanning window filter is implemented on the CCM such that the window length is optimized for a given frequency using a trial-and-error approach, following which the FCSM is obtained that is used to compute the DBF source map. The DBF algorithm is illustrated in Fig. 1(a), which shows a flow-chart illustrating its step-by-step implementation procedure, and the detailed working procedure is mentioned below.

(1) To begin with, the acoustic pressure data $p_{i}\left(t\right),i=1,2,...,N$ is recorded over a *N-*channel spiral microphone array, and the signals are converted to frequency-domain by computing its fast-Fourier Transform (FFT), i.e., $p_{i}\left(t\right)\overset{FFT}{\to }p_{i}\left(f_{0}\right).$
(2) Next, the CSM denoted by
  (1) $${\left[C(f_{0})\right]}_{N\times N}=\bar{p\left(f_{0}\right)p{\left(f_{0}\right)}^{H}},$$
  is computed, where $f_{0}$ denotes the frequency (Hz), the superscript *^H^* denotes the Hermitian or complex-conjugate transpose, and the superscript $\bar{X}$ denotes the averaging of the quantity *X* across a number of discrete time blocks using Welch's periodogram.
(3) The auto-spectra (microphone self-noise) given by the diagonal terms of the [**C**] matrix is set to zero, and the beamforming output $B(\xi ,f_{0})$ given by Eq. (2) is computed over a two-dimensional scanning grid or plane, and is called cross-spectral conventional beamforming (CBF) [13].
  (2) $$B\left(\xi ,f_{0}\right)=\frac{{\left\{g\right\}}_{1\times N}^{H}{\left[C\right]}_{N\times N}{\left\{g\right\}}_{N\times 1}}{{∥\left\{g\right\}∥}^{4}},$$
  where $\left\{g\right\}=e^{-jk_{0}r}/4\pi r$ denotes the free-space Green's function vector, also known as the steering-vector, and it implies wave propagation from an idealized monopole in a free-space without reflections or scattering. Furthermore, $r=|X_{n}-\xi_{s}|$, $X_{n}=\left(x_{n},y_{n},z_{n}\right)$**,** *n* = 1,2,...,*N* denote the locations of the *N* microphones, ξ denotes the grid-point co-ordinates on the scanning plane and $k_{0}=2\pi f_{0}/c_{0}=2\pi /\lambda_{0}$ represents the wavenumber while $c_{0}$ and $\lambda_{0}$ denotes the sound speed, and wavelength, respectively.

The predicted location is given by determining the maximum of the CBF source map. However, note that in semi-reverberant/reverberant environments or in situations where there is self-scattering from the surface of a source, such as an engineering application, there is likely to be a noticeable error in the predicted location. This is because the [**C**] matrix contains contributions from both direct and indirect (reflected and scattered) acoustic fields, and to improve the localization, it is necessary to filter out the indirect components.

(4) To start the windowing-based filtering technique, an inverse-FFT is used to compute the CCM from the CSM, i.e., ${\left[C(f_{0})\right]}_{N\times N}\overset{IFFT}{\to }{\left[C(\tau )\right]}_{N\times N},$ where τ denotes the quefrency domain.
(5) Plot the normalized auto-correlation graphs $C_{ii}\left(\tau \right)$, *i* = 1,2,...,*N* and identify the time-delay $\tau_{R,i}$ corresponding to the first reflection peak in each $C_{ii}\left(\tau \right)$ graph.
(6) Determine the minimum time-delay $\tau_{R,\min}=\min\left(\tau_{R,i}\right)$ corresponding to the first reflection peak considering the entire array. Note that $\tau_{R,\min}$ signifies the maximum time up to which the data is dominated by the direct field with the possibility of small reflection components. However, for $\tau >\tau_{R,\min},$ the data is contaminated with reflections and self-scattering as illustrated in Fig. 1(b).
(7) Next, define a Hanning window H(τ) for the time-interval $\tau =(-\tau_{R,\min},\tau_{R,\min})$ where the full-window $L_{w}$ equals $(2\tau_{R,\min}\times F_{s})-1$ data points, and the half-length $L_{hw}=\left(L_{w}-1\right)/2$ data points corresponding to the interval $\tau =[0,\tau_{R,\min}),$ where $F_{s}$ denotes the sampling frequency.
(8) Multiply each CCM time-vector $C_{ij}\left(\tau \right)$ with the Hanning window in $\tau =(-\tau_{R,\min},\tau_{R,\min})$ interval, i.e., $C_{ij}^{F}\left(\tau \right)=H\left(\tau \right)C_{ij}\left(\tau \right)$ where $C_{ij}^{F}\left(\tau \right)$ denotes the filtered-CCM (FCCM). Outside this window, i.e., for $\tau \leq -\tau_{R,\min}$ and $\tau \geq \tau_{R,\min},$ $C_{ij}\left(\tau \right)=0.$
(9) Compute the FCSM by implementing a FFT on FCCM, i.e., ${\left[C^{F}\left(\tau \right)\right]}_{N\times N}\overset{FFT}{\to }{\left[C^{F}\left(f_{0}\right)\right]}_{N\times N},$ which is used to compute the beamforming output using Eq. (2) where [C] matrix is replaced with $\left[C^{F}\right]$ matrix. The result is called the DBF source map, which will also exhibit a localization error because reflections cannot be completely filtered when the half-window length equals $L_{hw}.$ Nevertheless, this sub-optimal result will be used as a reference to compare with, and optimize the half-window length by further truncating the CCM in the next step.
(10) Determining the optimal half-window width $L_{ho}:$
(10.1) A sequence of Hanning window filter of half-lengths $L_{hi}=1,2,3,...,L_{hw}-1$ is defined, which is used to obtain the FCCM followed by computing the FCSM, which in turn is used to compute the sequence of parametric DBF source maps.
(10.2) Now, it is always desirable to have prior knowledge of the most probable location of the dominant source(s) for a real-world application. Keeping the same in-mind, one carefully analyzes through a *trial-and-error* procedure, the sequence of DBF maps for each $L_{hi}$ parameter based on which, the optimal half-window width $L_{hi}=L_{ho}$ is identified such that the localization error is minimized and simultaneously, the best focal-resolution is obtained, however, a trade-off generally exists between the localization error and resolution [15]. In practice, a sensitivity analysis about the optimal half-window width $L_{ho}$ is carried out to determine a narrow range of half-window lengths, typically within the interval $L_{hi}=\left[L_{ho}-5,L_{ho}+5\right]$ points such that the small uncertainty in the predicted location is $\lambda_{0}/20$ or less.
(10.3) The analysis of parametric DBF maps to identify $L_{ho}$ is based on the frequency of interest, i.e., the wavelength versus the noise source dimensions, and broadly, two frequency regions are identified:

(a) Low-frequency region where the wavelength is much larger than the noise source dimensions. Here, it is empirically found that $L_{ho}$ consists of only the first few data points, i.e., $L_{ho}\ll L_{hw}.$ An increase in the data points beyond the upper-limit of the interval produces a noticeable increase in localization error and generation of side-lobes, similar to the CBF maps.
(b) Mid- and high-frequency regions where the wavelength is either comparable to, or greater than the noise source dimensions, respectively. It is again empirically observed that $L_{ho}$ consists of several data points, which is necessary to retain sufficient data to produce accurate results, and its width is comparable to $L_{hw}$. Here, if only the first few data points are considered, it causes an excessive loss of the direct field data, thereby resulting in a counterproductive result - the DBF map is then shown to exhibit only the arms of the spiral array, which manifest as dominant side-lobes while the main source will no longer be visible.

### CLEAN-SC deconvolution to enhance the resolution of DBF source maps

The source resolution of the DBF maps is improved by implementing a deconvolution CLEAN-SC algorithm, where the acronym SC stands for *Source Coherence,* meaning that all the sources which are coherent with the main lobe are removed from the acoustic images, see Sijtsma [4]. The underlying idea is to first search for the maximum source auto-power, i.e., the predicted location in the DBF or ‘dirty’ map. Following this, the point spread function (PSF) is computed using the measured acoustic data, which is subtracted from the DBF map. The process is implemented iteratively, thereby successively removing regions from the DBF map that are spatially coherent with the main lobe, which allows precise extraction of the absolute sound pressure levels from the dirty maps and enables the side-lobe suppression. The main steps involved are briefly summarized [4].

(1) Apply the DBF algorithm mentioned above to compute the beamforming output over a scanning plane, which is referred to as the ‘dirty’ map. The peak location $\xi_{\max}$ corresponding to a dominant source is identified, and the corresponding FCSM $\left[C_{\max}^{F}\right]$ is then given by
  (3) $$\left[C_{\max}^{F}\right]=\frac{{\left[C^{F}\right]}_{N\times N}{\left\{w_{\max}\right\}}_{N\times 1}{\left\{w_{\max}^{*}\right\}}_{1\times N}{\left[C^{F}\right]}_{N\times N}}{A_{\max}},$$
  where ${}^{*}$ denotes complex-conjugate transpose, $A_{\max}=\left\{w_{\max}^{*}\right\}\left[C^{F}\right]\left\{w_{\max}\right\}$ is the maximum amplitude and weight-vector $\left\{w_{\max}\right\}=\frac{\left\{g_{\max}\right\}}{{∥\left\{g_{\max}\right\}∥}^{2}}$ evaluated at a dominant source location $\xi_{\max}.$
(2) Replace $\left[C^{F}\right]$ with $\left[C^{F}\right]-\phi \left[C_{\max}^{F}\right],$ where ϕ is the safety factor known as loop gain, and it must be within 0<ϕ≤1. In this work, following Refs. [4,23], we set ϕ=0.9.
(3) Repeat step 1 unless the stopping criterion $\text{norm}\left(\left[C^{F}\right]-\phi \left[C_{\max}^{F}\right]\right)\geq \text{norm}\left(\left[C^{F}\right]\right)$ is satisfied.
(4) The summation of $\left[C_{\max}^{F}\right]$ matrices obtained after every iteration, i.e., $\sum_{i=1,2,...}{\left[C_{\max}^{F}\right]}_{i}$ is obtained, which is used to compute the CLEAN-SC map.

In this manner, the DBF + CLEAN-SC map can be computed, which can deliver accurate location and improved resolution of a constant beamwidth for multiple source(s) placed in reverberant or semi-reverberant environments.

### Acoustic instrumentation and data acquisition system (DAQ)

The acoustic instrumentation consists of a planar microphone array of 31 G.R.A.S. 40PH ¼” microphones, which were connected to 4497 PXIe National Instruments (NI) DAQ cards using BNC cables. These DAQ cards were mounted on a 1073 NI PXIe chassis. The data acquisition system was linked to a high-performance desktop computer featuring an 11th generation i7 processor and 32 GB of RAM, while the signals were acquired through a LabView program. The microphone array follows a standard Underbrink spiral design, known for its satisfactory performance across both low and high frequencies [24]. To calculate the microphone locations, we first select the maximum and minimum radii of the spiral, which is denoted by $r_{\max}$ and $r_{\min}$, respectively, the number of spiral arms $N_{a}$, the number of microphones per spiral $N_{m}$, and the spiral angle ν. The area of the array is then separated into *N*_m_ - 1 equal area annuli, which are further subdivided into equal area segments, and microphones are placed at the center of these segments. Furthermore, an inner circle of microphones is added at $r_{\min}$ to improve the high frequency performance, and an extra microphone is placed at the origin. The radial positions of the microphones are given by

(4a) $$r_{m,1}=r_{\min},m=1,2,...,N_{a},$$

(4b) $$r_{m,n}=\sqrt{\frac{2n-3}{2N_{r}-3}}r_{\max},m=1,2,...,N_{a},n=2,3,...,N_{m}.$$

The angular positions are calculated by placing each microphone along a log spiral, and rotating the spiral around the origin so that there are *N*_a_ spiral arms. Thus, the angular co-ordinate of the microphone in the *m*^th^ spiral arm at *n*^th^ location is given by

(4c) $$\theta_{m,n}=\frac{\ln\left(\frac{r_{m,n}}{r_{\min}}\right)}{\cot\nu }+\left(2\pi \frac{m-1}{N_{a}}\right),$$

**Fig. 2:**
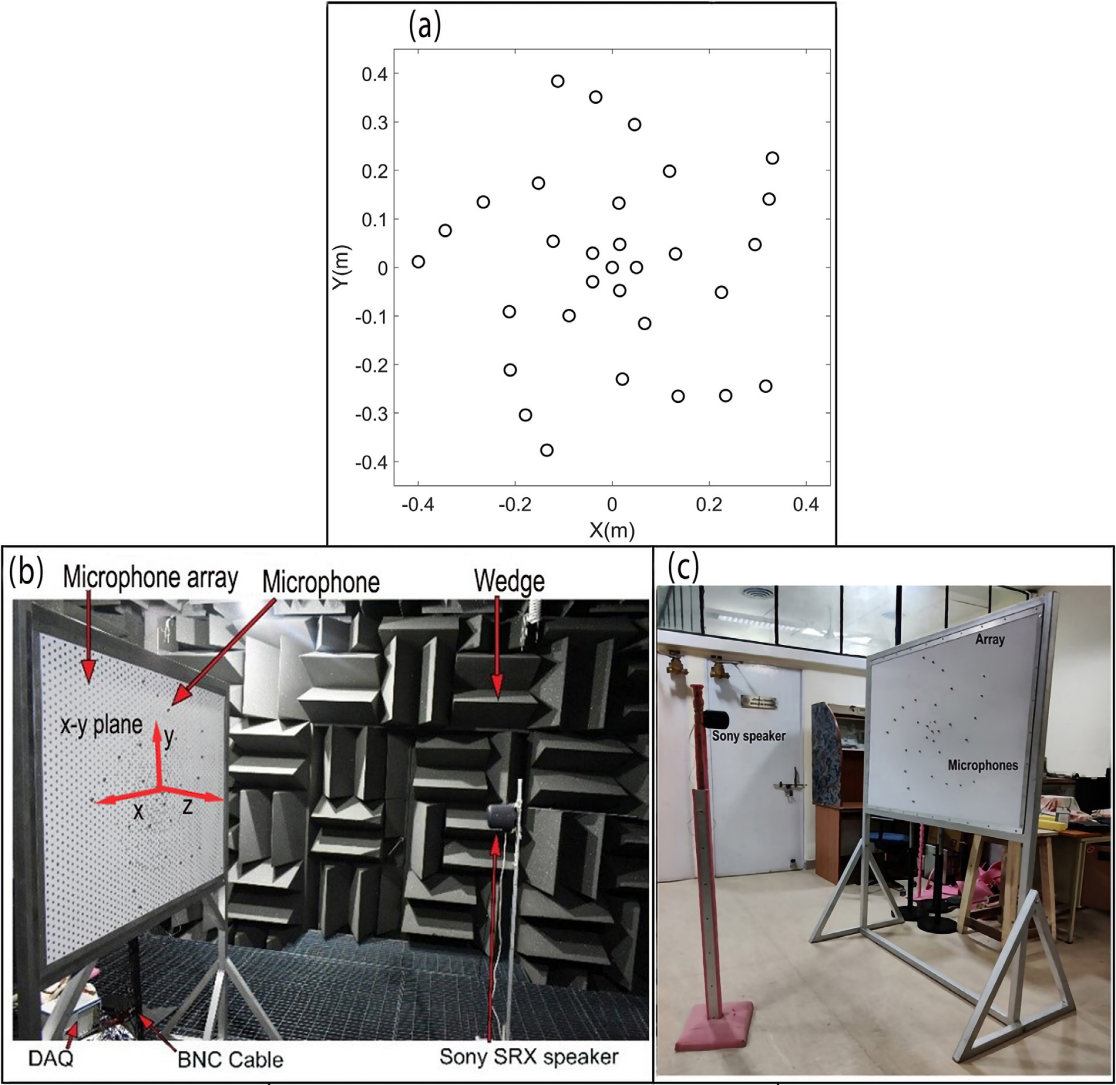
(a) Distribution of microphones arranged in a 31-channel Underbrink array. Parts (b) and (c): Photographs of the experimental set-up of a loudspeaker source placed in an anechoic chamber (part (b)) and in a semi-reverberant room (part (c)).

In this work, $r_{\max}=500\;\text{mm}$ and $r_{\min}=50\;\text{mm},$thus, the array aperture was equal to 1 m. The spiral angle ν=5π/16, while five spiral arms and six microphones in each arm, was considered, and a microphone was also placed at the center. Fig. 2(a) shows the spatial locations of the microphones in the Underbrink spiral array configuration considered here. The microphone array and DAQ system were used to record the noise data at sampling rate $F_{s}=65536\;\text{Hz}$ and sample time *T =* 10 s.

### Test-cases for demonstrating the DBF in anechoic and semi-reverberant room environments

#### Loudspeaker source

The test-case of a small loudspeaker is considered because it is the simplest controlled source. A SONY SRS-XB12 speaker with a diameter of 75 mm and an effective operating frequency range of 100 to 7000 Hz was used. Experiments were first conducted in an anechoic chamber, and the CBF source maps obtained will be used as a reference to compare with the DBF source maps obtained in the semi-reverberant room.

Fig. 2(b) shows the loudspeaker mounted on a steel stand and placed in an anechoic chamber, which is made of sound-absorbing polyurethane foam wedges of height 450 mm and an included half-angle $\theta =18.4^{\circ }.$ Its working dimensions, i.e., tip-to-tip distance between wedges on the opposite walls are 5 m x 5 m x 3 m. The chamber provides a reflection-free environment above 200 Hz, and the background noise was 40 dB(A). The loudspeaker was placed in front of the spiral array-oriented along the *x-y* plane, and the perpendicular distance between the array plane and the source was fixed at *z =* 1.5 m. The coordinates of the center microphone were taken as the origin (0, 0, 0), while the center of the speaker was located at (0, 0, 1.5 m). Fig. 2(c) shows the same loudspeaker source mounted on a wooden stand and placed at the same distance *z =* 1.5 m in front of the microphone array in the semi-reverberant room environment that has multiple hard scattering and/or reflecting surfaces. The loudspeaker was made to emit bandlimited white noise signals in one-third octave bands centered at $f_{c}=500\;\text{Hz},1000\;\text{Hz}$ and 2000 Hz, respectively, and the data was recorded on the microphone array.

In both anechoic chamber and the semi-reverberant room environment, the ambient temperature was measured to be $28^{\circ }C,$ which corresponds to a sound speed $c_{0}=347.8m\cdot s^{-1}.$

#### Mixer-grinder home appliance

**Fig. 3:**
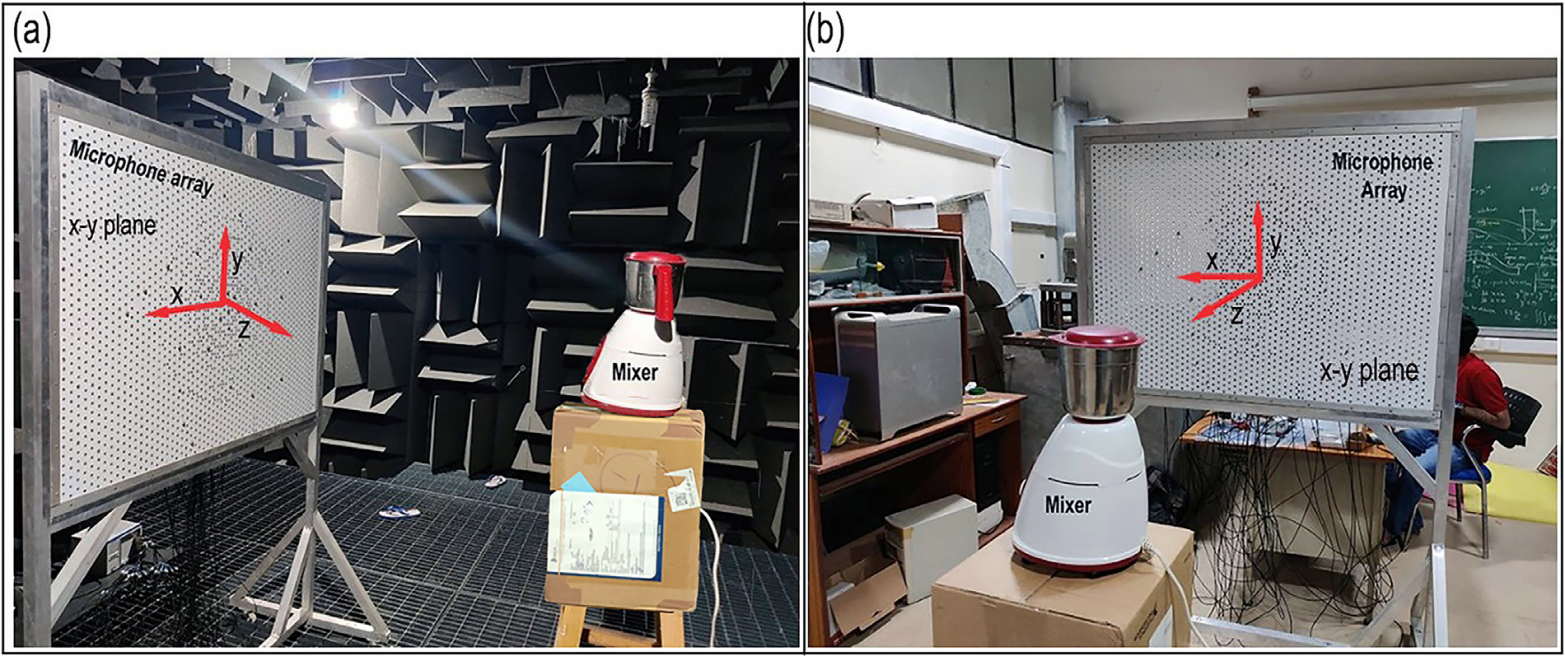
Photographs of the experimental set-up of a mixer-grinder home-appliance placed (a) in an anechoic chamber and (b) in a semi-reverberant room.

Fig. 3(a) and (b) show the photograph of a Bajaj GX 3701 mixer-grinder home appliance located in (a) the anechoic chamber and (b) the same semi-reverberant room space.

The power rating of the mixer-grinder was 750 W, and it was equipped with three heavy-duty stainless steel (SS) grinding blades that were designed for a maximum speed of 18,000 RPM. The mixer-grinder, however, can also be operated at lower speeds of 12,000 and 6000 RPM. The white-coloured hub houses the electrical motor, which drives the mixer-grinder system and the hub height was 325 mm while its top diameter was 210 mm. The height of the 3-litre steel jar in which grinding takes place was roughly 200 mm. As before, the origin (0, 0, 0) was fixed at the center of the spiral array, and it was aligned with the mounting interface of the grinding jar with the hub whose co-ordinates *w.r.t.* the origin are given by (x=0,y=0,z=1.5m). Therefore, in both environments, data was recorded on the Underbrink spiral array, which was oriented along the plane located at a distance *z =*1.5 m from the center of the mixer-grinder hub. The radiated far-field noise is attributed pre-dominantly to the electric motor and power transmission gearbox located inside the hub as well as the grinding blades, which rotate at the bottom of the jar – these noise producing components are located in-proximity. Therefore, the small region near the top of the hub or just below the mounting center of the grinding blades was assumed to be the approximate known location of the noise source.

### Acoustic spectra of the mixer-grinder

**Fig. 4:**
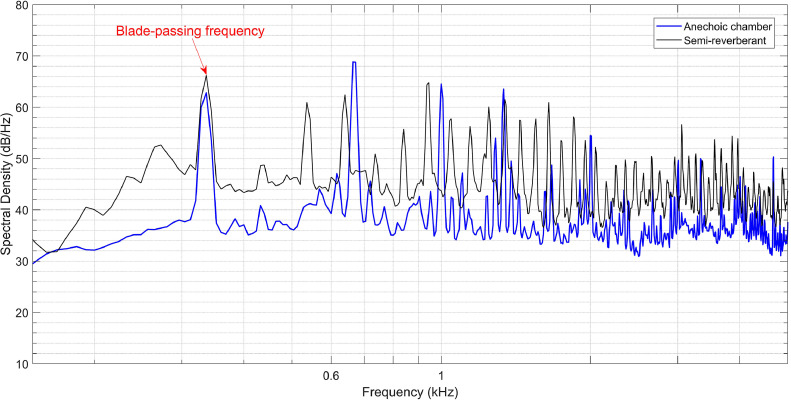
Acoustic spectrum of the mixer-grinder measured in the anechoic chamber and semi-reverberant room environment. The fundamental blade-passing frequency (BPF) has been annotated.

Fig. 4 shows a comparison of the acoustic spectrum of the mixer-grinder home appliance measured at the center microphone of the array located in the anechoic chamber with that obtained in the semi-reverberant room when it was operated at its lowest operating speed, i.e., at 6000 RPM.

The spectra measured in both anechoic and semi-reverberant environments exhibit a broadband nature with multiple tones, which mainly occur at the integer multiples of blade passing frequency (BPF) of the rotating blades located within the grinder. In comparison to the anechoic chamber, the spectrum measured in the room environment is approximately 7 to 8 dB higher due to multiple reverberations and exhibits additional tones. The first resonance peak or tone in the acoustic spectrum is expected to occur at the first BPF given by (3×6000)/60=300Hz, however, it was observed at 336 Hz in both anechoic and room environments. The higher harmonics occur at its integer multiples, such as 672 Hz, 1008 Hz and 1344 Hz, and so on in the anechoic chamber. On the other hand, in the semi-reverberant environment, the super-harmonics slightly deviate from integer multiples, and one observes peaks at 640 Hz, 960 Hz, and 1352 Hz, so on.

## Method validation

### Loudspeaker source

**Fig. 5:**
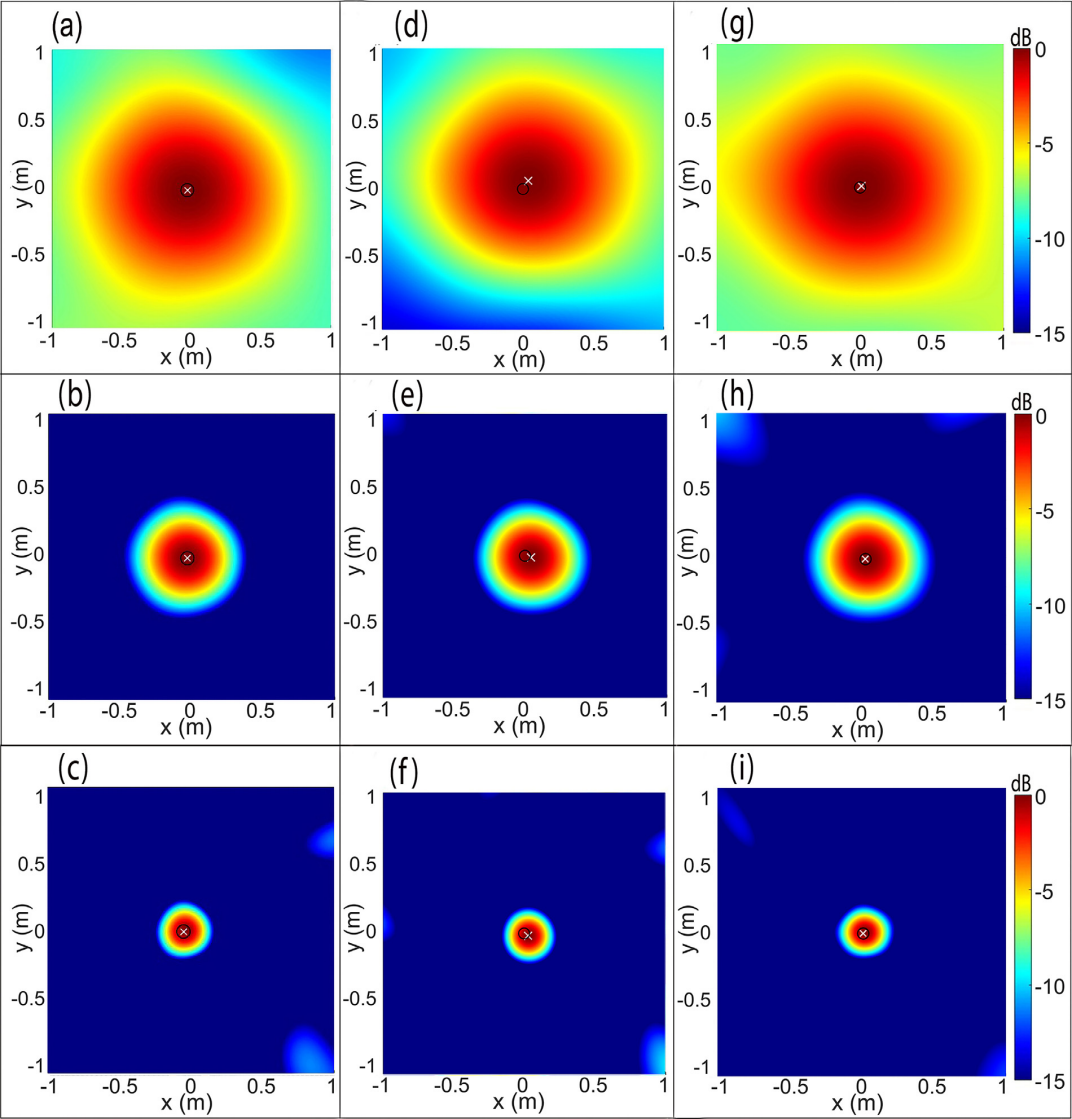
Parts (a-c): CBF source maps of the loudspeaker placed in an anechoic chamber array at tonal frequencies 500 Hz, 1000 Hz, 2000 Hz. Parts (d-f): CBF source maps of the loudspeaker placed in the semi-reverberant room, at the same frequencies, respectively. Parts (g-i): Counterpart DBF source maps of the loudspeaker placed in the semi-reverberant room computed using optimal windowing of the CCM signals.

Fig. 5(a–c) and Fig. 5(d–f) presents the CBF maps of the small loudspeaker source placed in the anechoic chamber and semi-reverberant room environment, respectively, computed at the center frequency of the one-third octave bands mentioned earlier and in that order. The maps were normalized with respect to the focal-point, i.e., the source strength and the results are shown over a dynamic range [0, -15 dB]. As anticipated, Fig. 5(a–c) show that in an anechoic chamber, the CBF can accurately the loudspeaker, which is represented by the circular focal-spot. The predicted location, i.e., the focal point (0 dB) denoted by a cross **X** is observed to be co-incident with the known source location, which is indicated by a circle **O** taken at the center of the circular cross-section of the loudspeaker. The same dynamic range and symbolic conventions are used for the remaining source maps presented in this work.

**Fig. 6:**
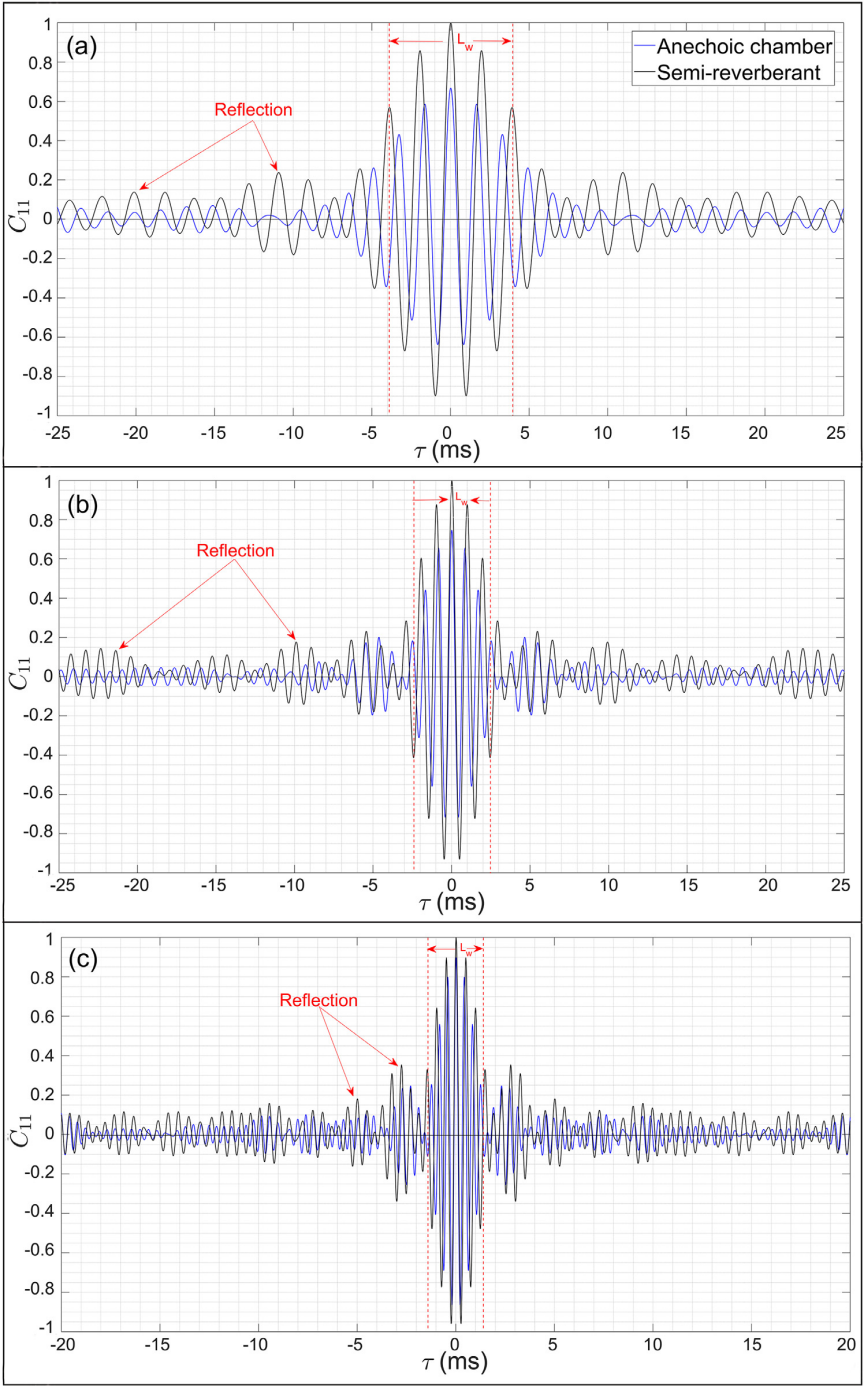
Auto-correlation function $C_{11}\left(\tau \right)$ computed at the center microphone for the loudspeaker source emitting tonal noise at frequencies (a) $f_{0}=500\;\text{Hz},$ (b) $f_{0}=1000\;\text{Hz},$ and (c) $f_{0}=2000\;\text{Hz},$ in the anechoic chamber and the reverberant room.

Fig. 5(d–f), on the other hand, show that in a semi-reverberant room, the CBF delivers a satisfactory performance inasmuch as the source map can be readily interpreted from the nearly circular focal spot, notwithstanding a small localization error given by $0.1\lambda_{0},$$0.13\lambda_{0}$ and $0.19\lambda_{0}$ at 500 Hz, 1000 Hz, and 2000 Hz, respectively, which is attributed to reflections and scattering from the surroundings. The DBF algorithm is now implemented to improve the localization. To this end, the normalized auto-correlation graphs $C_{11}\left(\tau \right)$ pertaining to the center microphone are presented in Fig. 6(a–c) for $f_{0}=\left\{500,1000,2000\right\}\;\text{Hz},$ respectively, which are symmetric with respect to quefrency τ. The occurrence of reflections can be readily observed from the repeating wave packets pattern whose peak amplitude progressively decreases with τ. The peak due to the direct field occurs at τ=0 while the first reflection occurs at time-delay $\tau_{R}\approx 10.87\;\text{ms},5.44\;\text{ms},$ and 2.76ms, in Fig. 6(a–c), respectively, thereby indicating the time τ beyond which the field at the center microphone is definitely contaminated by reflections. The CCM graphs $C_{ii}\left(\tau \right)$ for each microphone in the array were analyzed to determine the time-interval $\tau =[0,\tau_{R,\min})$ for which the direct field dominated the acoustic field. For $f_{0}=\left\{500,1000,2000\right\}\;\text{Hz},$ the time $\tau_{R,\min}$ was found equal to 3.87ms, 2.51ms, and 1.32ms, respectively, for which, the corresponding $L_{hw}$ was given by the first 253, 164 and 86 data points, respectively. Next, a sequence of source maps was computed corresponding to the half-lengths $L_{hi}=1,2,3,...,L_{hw}-1$ to determine the optimal half-window length $L_{ho}$ for each frequency considered. It was found that when $L_{ho}=\left\{5,34,60\right\}$ data points were considered for $f_{0}=\left\{500,1000,2000\right\}\;\text{Hz},$ respectively, the predicted location was co-incident with the loudspeaker center, i.e., localization error was zero as observed from the DBF maps presented in Fig. 5(g–i), respectively. Furthermore, note that optimal half-window length increases with frequency – only the first few data points are needed at low frequencies, while many more points should be considered at higher frequencies to retain sufficient direct field data.

In light of the above discussion, one may conclude that even for the test-case of a small loudspeaker source placed in a semi-reverberant environment, it is recommended to implement the CCM windowing-based DBF algorithm to remove the marginal localization error. In contrast, the counterpart $C_{11}\left(\tau \right)$ graphs for the loudspeaker in the anechoic chamber presented in Fig. 6(a–c) is purely due to the direct field as observed from the gradual decay of oscillations, which makes the implementation of a Hanning window unnecessary.

### Mixer-grinder home-appliance

#### Anechoic chamber

**Fig. 7:**
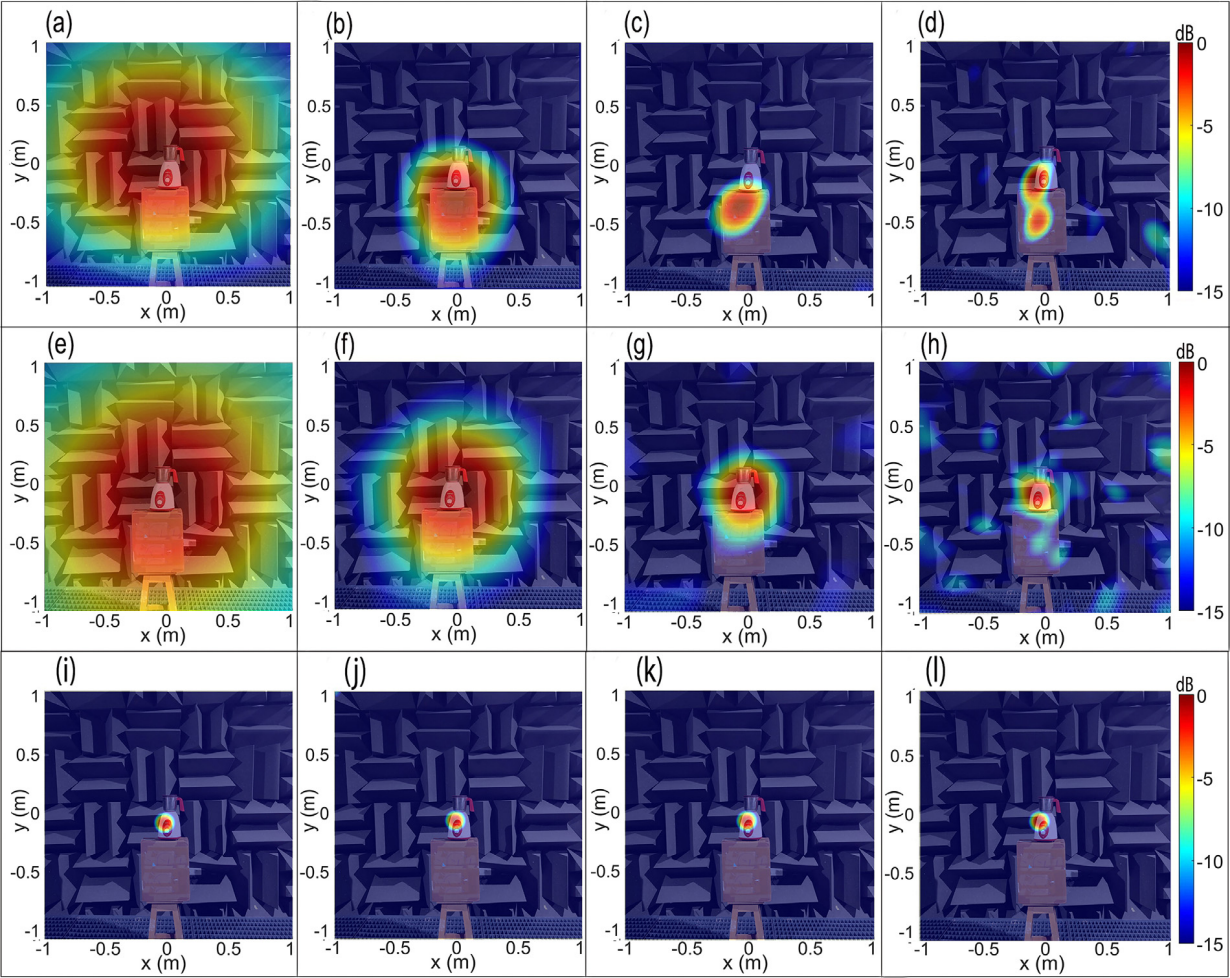
Beamforming maps of mixer-grinder placed in the anechoic chamber at f=672Hz,f=1008Hz,f=2008Hz, and f=4680Hz. Parts (a-d) show the CBF maps, parts (e-h) show the DBF maps while parts (i-l) present the DBF+CLEAN-SC maps at the aforementioned frequencies (in that order).

Fig. 7(a–d) show the CBF maps of the mixer-grinder home appliance in the anechoic chamber at tonal frequencies (a) $f_{0}=672\;\text{Hz},$ (b) $f_{0}=1008\;\text{Hz},$ (c) $f_{0}=2008\;\text{Hz},$ and (d) $f_{0}=4680\;\text{Hz},$ respectively, computed on a scanning plane passing through the mounting interface of the grinding jar with the hub, and located at a distance *z =*1.5 m from the spiral array plane. The mixer-grinder photograph is watermarked in the background, which makes it easy to interpret the source map.

Fig. 7(a) shows that at 672 Hz, the source resolution is very poor, although one can appreciate that the mixer-grinder is the noise source. At higher frequencies, parts (b-d) indicate that the focal-spot location is slightly offset from the hub of the mixer-grinder, which is the anticipated location (noted earlier). Indeed, the focal-spot center in Fig. 7(b) and (c) were localized well-below the mixer-grinder hub, more precisely, on the supporting cardboard box, while in Fig. 7(d), the focal-spot center was localized near the vicinity of the hub, which suggests a small error and the occurrence of a side-lobe which can make it challenging to identify the actual location. These results indicate that even in an anechoic environment, it is difficult to record the pure direct field emitted by an engineering application such as mixer-grinder because the data recorded at the array may indeed be contaminated with the reflected field components attributed to self-scattering by the mixer-grinder body and to a much lesser extent, from the array surface.

It is therefore, evident that the CBF is likely to produce a noticeable error in localizing the noise source(s) generated from an engineering application even when it is tested in an anechoic chamber due to the self-scattering of waves from the mixer-grinder surface and it then becomes necessary then to implement the DBF to resolve the issue.

### Implementation of the optimized half-window filter DBF algorithm

**Fig. 8:**
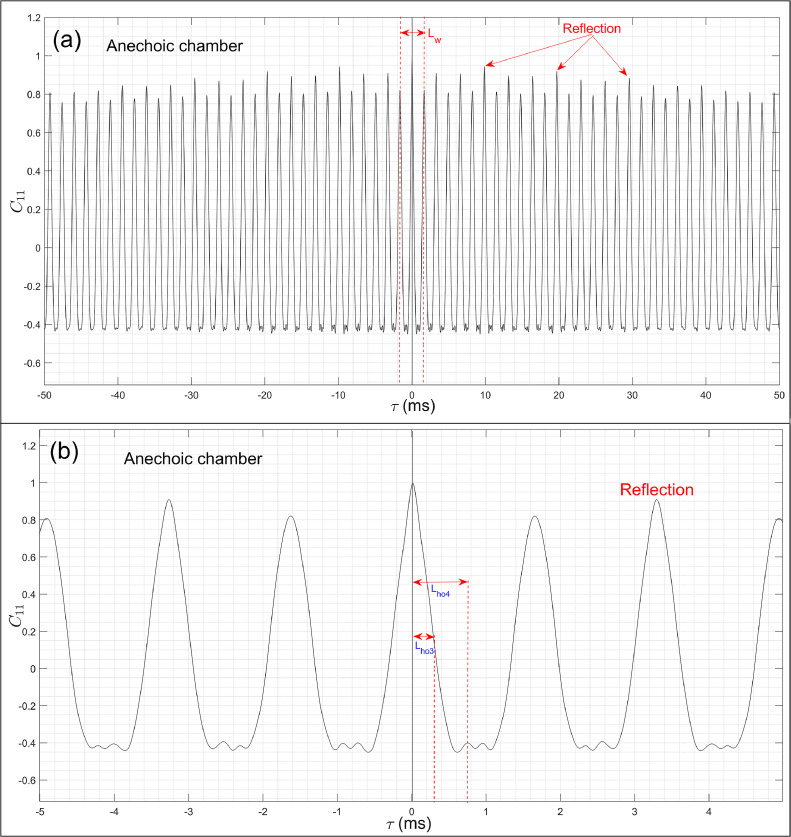
Auto-correlation graph $C_{11}\left(\tau \right)$ of the mixer-grinder obtained in the anechoic chamber. Part (a) presents an overall view over a larger time-duration in which the reflection peaks have been annotated while part (b) presents an exaggerated view over a shorter time-duration. The optimal half-window lengths for different frequencies have been marked in part (b).

Fig. 8(a) and (b) present the normalized auto-correlation graph $C_{11}\left(\tau \right)$ of the mixer-grinder obtained in the anechoic chamber – part (a) presents an exaggerated view over the time-duration τ=[−5,5]ms while part (b) shows the overall view over τ=[−100,100]ms. Fig. 8(b) shows that in the anechoic chamber, $C_{11}\left(\tau \right)$ graph oscillates and its amplitude decays very slowly with time. Indeed, a closer look indicates the presence of multiple reflections occurring at regular intervals whose magnitudes are marginally higher than the main signal, which possibly explains the small localization error observed in Fig. 7(b–d). The first reflection in $C_{11}\left(\tau \right)$ graph is annotated in Fig. 8(b), which occurs at $\tau_{R}=3.28\text{ms},$ however, considering all 32 microphones, the minimum time at which the first reflection occurs equals $\tau_{R,\min}=$ 1.63 ms, which corresponds to $L_{hw}=$107 data points.

**Fig. 9:**
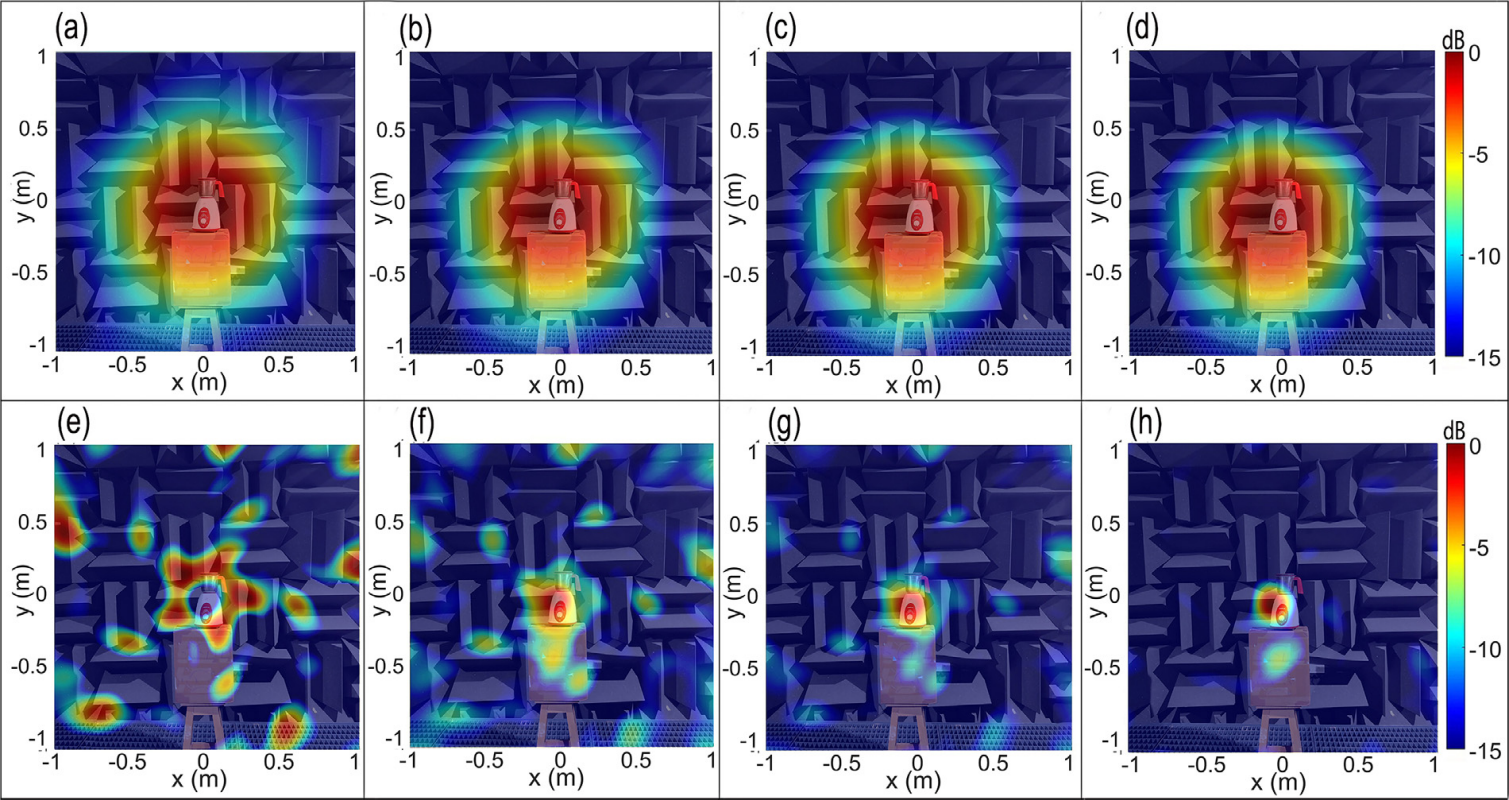
Parametric study to analyze the effect of half-window length $L_{hi}$ on the DBF source maps of the mixer-grinder placed in the anechoic chamber. Parts (a-d) show the DBF maps for $f_{0}=$ 1008 Hz obtained by considering $L_{hw}=$ 10, 20, 50, and 100 data points, respectively, while parts (e-h) show the DBF maps for $f_{0}=$4680 Hz by considering $L_{hw}=$ 10, 20, 50, and 100 data points, respectively.

To determine the frequency dependent optimal half-window width, a trial-and-error procedure is followed, which is based on minimization of the localization error, i.e., it is aimed to obtain the focal-spot center closest to the region near the top of the hub, which is the approximate known location of the noise source. As before, the DBF source maps were computed by successively increasing the half-window lengths $L_{hi}=\left\{1,2,...,n\right\}$ and the results of this parametric study are presented at selected values of $L_{hi}=$ 10, 20, 50 and 100 data points for $f_{0}=1008\;\text{Hz}$ in Fig. 9(a–d), respectively, and for higher frequency $f_{0}=4680\;\text{Hz}$ in Fig. 9(e–h), respectively. Fig. 9(a) shows that the best localization was obtained when $L_{hi}=L_{ho2}=10$ data points, however, when more points were considered, the focal-spot center gradually shifted from the desired location as observed from Fig. 9(b–d), thereby exhibiting a progressively increasing error. From Fig. 9(e) and (f), it was observed that choosing a small half-window given by 10 and 20 points results in excessive loss of the direct field data, and instead of delivering the focal-spot centered at the desired source location, the DBF map exhibits only the arms of the spiral array which manifests as dominant side-lobes. Fig. 9(g) shows that when $L_{hi}=L_{ho4}=$ 50, the focal point was nearly co-incident with the most probable source location while a further increase in the half-window tends to produce a small localization error, refer to Fig. 9(h). The parametric study was also carried out for $f_{0}=672\;\text{Hz}$ and 2008 Hz, wherein the optimal half-window width was found equal to $L_{ho1}=5$ and $L_{ho3}=20,$ respectively. While $L_{ho3}$ and $L_{ho4}$ have been annotated in Fig. 9(a), $L_{ho1},$$L_{ho2}$ are not indicated due to the time-window chosen on the *x*-axis. Furthermore, a sensitivity analysis was carried out for each frequency considered here, which revealed that the focal point location does not exhibit a noticeable variation ($\lambda_{0}/20$ or less) with small changes around the optimal half-window length, i.e., the resultant DBF maps were not sensitive to a small variation around the optimal window value.

Fig. 7(e–h) presents the counterpart DBF source maps computed using the frequency-dependent optimal half-window lengths noted above. It is observed that the nearly circular focal-spot is now centered at a region near the top of the hub just below the location of grinding blades, i.e., the predicted location is co-incident with the approximate known location, which suggests that it is important to implement the DBF algorithm even in an anechoic chamber to eliminate self-scattering from the body of an engineering application that tends to produce a noticeable localization error. Fig. 7(e) and (f), show that at low frequencies, the resolution is poor due to the large size of the focal-spot. Fig. 7(g) and (h) also show that the focal spot is centered near the top of the hub, i.e., they demonstrate a readily noticeable improvement in the source localization as compared to their counterpart results presented in Fig. 7(c) and (d), respectively. Now, considering the predicted source location obtained in the DBF maps shown in Fig. 7(e–h) as a reference, the non-dimensional localization error or deviation of the focal point in the CBF maps obtained in Fig. 7(a–d) is quantified in Table 1, which shows that the error increases with an increase in frequency. Furthermore, the source resolution in Fig. 7(g) and (h) is far better than Fig. 7(e) and (h), which can be explained by noting that the resolution improves, i.e., focal-spot size decreases with at higher frequencies. Here, the focal-resolution was taken equal to Full-Width at Half-Maximum (FWHM) given by the sum of the distances measured along a straight line passing through the focal point and corresponds to -6 dB level on either sides [25]. The resolution was measured along four different lines making an angle θ=(0,π/4,π/2,3π/4) with the *x*-axis and passing through the focal point [14,15]. The average denotes the focal-resolution, which is given by $3.49\lambda_{0},2.54\lambda_{0},2.42\lambda_{0}$ and $2.68\lambda_{0}$ in Fig. 7(e–h), respectively, where $\lambda_{0}$ is the corresponding wavelength.

**Table 1:** Non-dimensional localization error of the predicted location in the CBF maps of the mixer-grinder home appliance with respect to the counterpart DBF maps.

**Frequency (Hz)**	672	1008	2008	4680
**Non-dimensional localization error**$\Delta /\lambda_{0}$	0.09	0.54	1.42	2.0

The CLEAN-SC deconvolution algorithm is now implemented to improve the focal-resolution of the DBF maps of the mixer-grinder noise source. To this end, the loop gain parameter φ in the iteration process was chosen equal to 0.99, and for all frequencies considered, the clean beamwidth was set to 50 mm at 3 dB below the peak levels [4,23]. Fig. 7(i–l) show a dramatic improvement in the source resolution, particularly at lower frequencies – the implementation of CLEAN-SC is shown to completely suppress the regions of focal spot, which are coherent with the main lobe, and the clean beams now clearly reveal the dominant noise source region localized at the top of the mixer-grinder hub.

#### Semi-reverberant room environment

**Fig. 10:**
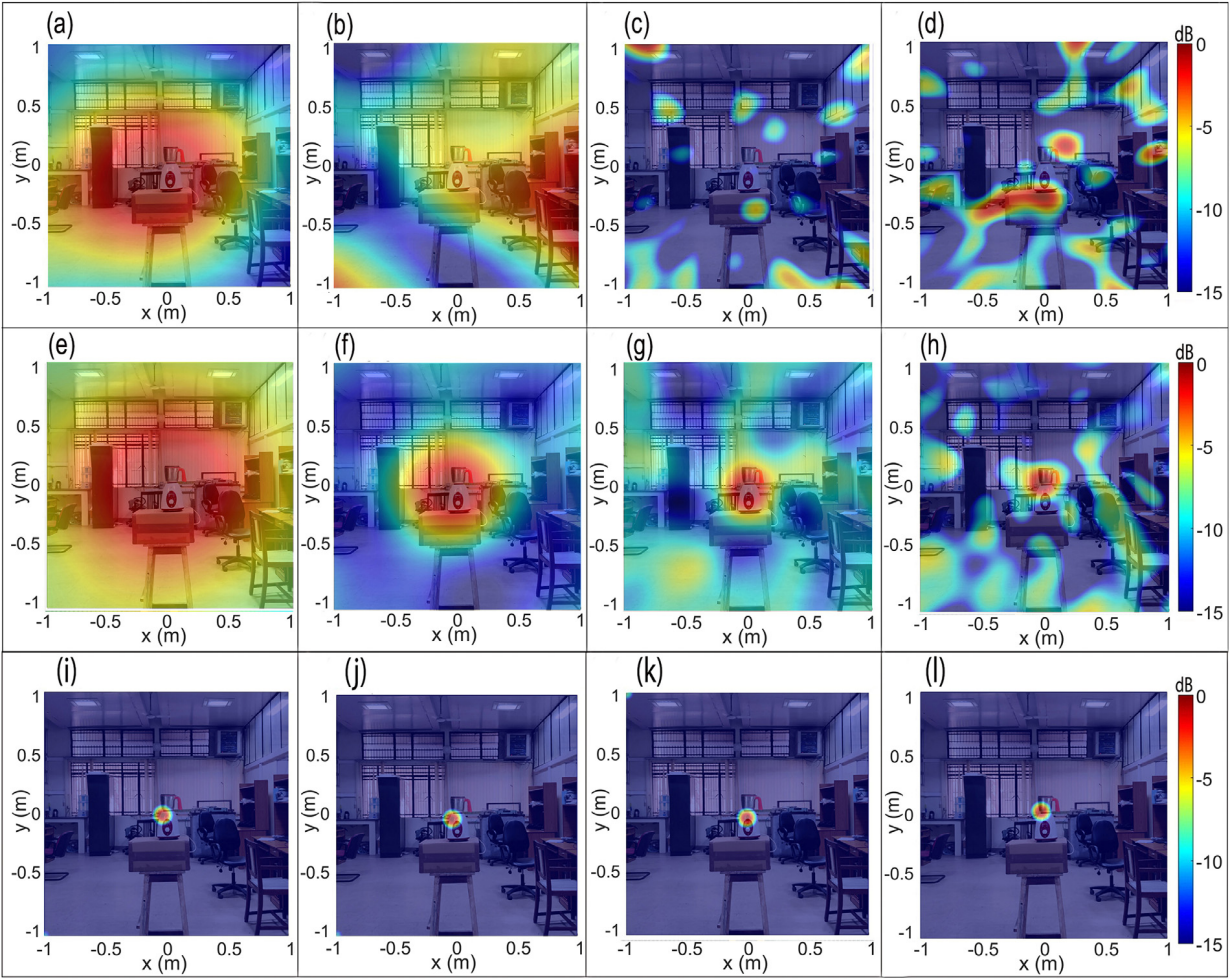
Beamforming maps of mixer-grinder placed in the semi-reverberant room at f=640Hz,f=960Hz,f=2000Hz, and f=4672Hz. Parts (a-d) show the CBF maps, parts (e-h) show the DBF maps while parts (i-l) present the DBF+CLEAN-SC maps at the aforementioned frequencies (in that order).

Fig. 10(a–d) show the CBF source maps of the mixer-grinder home appliance placed in the semi-reverberant room environment computed for frequencies (a) $f_{0}=$ 640 Hz, (b) $f_{0}=$ 960 Hz, (c) $f_{0}=$ 2000 Hz, and (d) $f_{0}=$ 4672 Hz, computed on a scanning plane located at a distance *z =*1.5 m from the array plane. Fig. 10(a) exhibits a large focal-spot size that completely overwhelms the mixer-grinder, exhibiting poor resolution. Additionally, note that the focal-spot is slightly offset from the source location, which indicates a small localization error. Fig. 10(b–d) suggest that with an increase in frequency, the CBF method completely fails to localize the source, which is attributed to a significant reverberation of the mixer-grinder signals in the room environment. Note that the focal spot representing the source is absent in Fig. 10(b–d), instead, large side-lobes occur, which makes it almost impossible to interpret the source maps.

### Implementation of the optimized half-window filter DBF algorithm

**Fig. 11:**
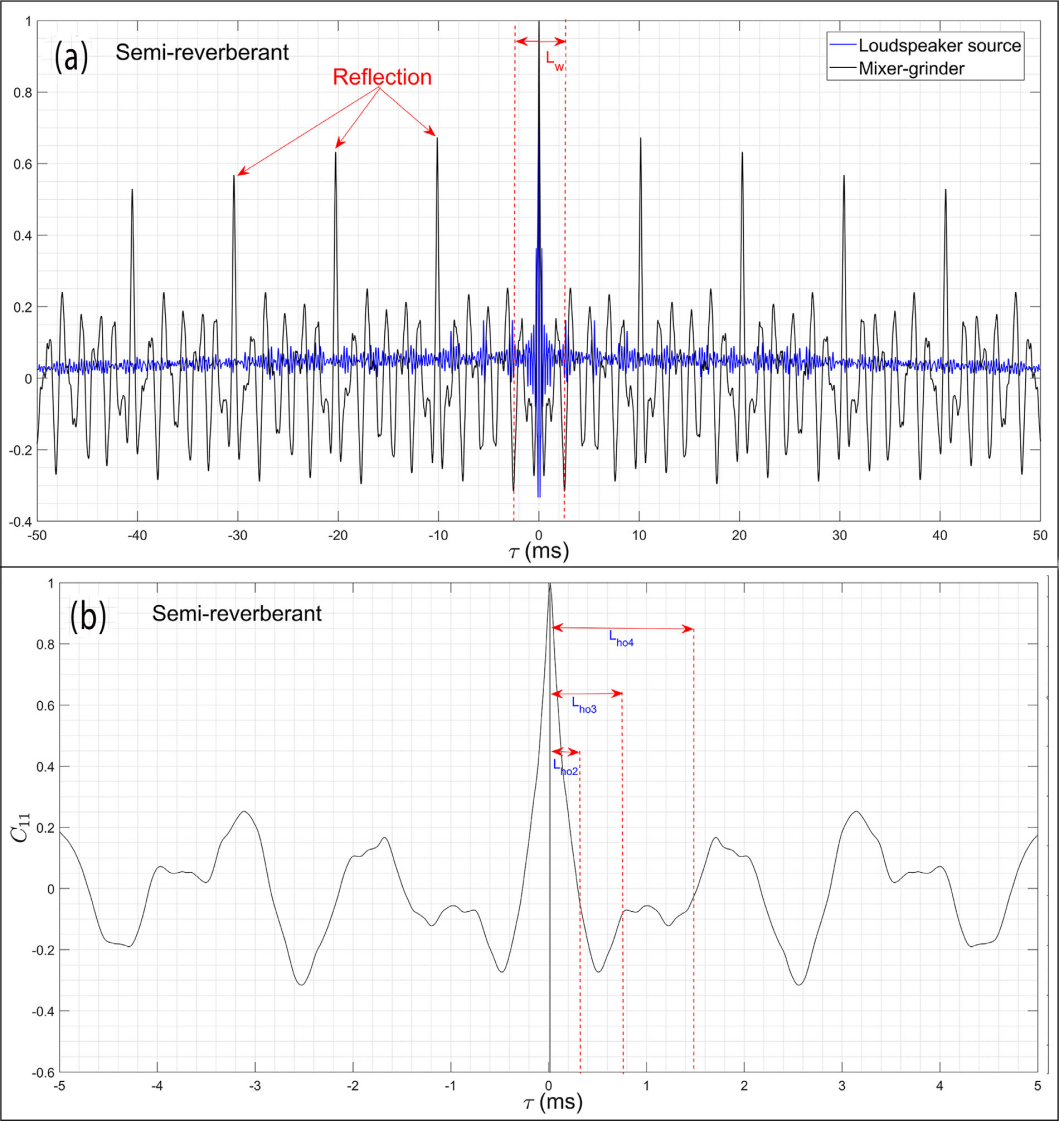
Auto-correlation graph $C_{11}\left(\tau \right)$ of the mixer-grinder and simple loudspeaker source obtained in the semi-reverberant room. Part (a) presents an overall view over a larger time-duration in which the reflection peaks have been annotated for the mixer-grinder while part (b) present an exaggerated view over a shorter time-duration in which the optimal half-window lengths for different frequencies have been marked.

To minimize the localization error, the DBF algorithm was implemented. To this end, we consider the $C_{11}\left(\tau \right)$ graph in the semi-reverberant room space presented in Fig. 11(a) and (b) – parts (a) and (b) show an exaggerated view and overall view of the auto-correlation graphs over the time-duration τ=[−5,5]ms and τ=[−25,25]ms, respectively. A comparison of the $C_{11}\left(\tau \right)$ graph in Fig. 11(a) with Fig. 8(a) readily demonstrates the significant influence of the semi-reverberant environment on the auto-correlation waveform. Fig. 11(b) shows that the first reflection occurs at $\tau_{R}=10.11\;\text{ms},$ however, considering all 32 microphones, it was found that $\tau_{R,\min}$ = 2.56 ms or the first 168 data points. Following a trial-and-error approach as before, the approximate optimal half-window lengths at the noted frequencies were found to be equal to $L_{ho1}=10,$ $L_{ho2}=20,$ $L_{ho3}=50,$ and $L_{ho4}=100$ data points, respectively, which are double their counterpart values obtained in the anechoic chamber.

Fig. 10(e–h) presents the counterpart DBF maps computed using the frequency-dependent optimal half-window lengths noted above. While Fig. 10(e) suffers from poor source resolution, which is typical at low-frequencies, the focal-spot is now centered at the source location just below the top of the hub, thereby indicating an improvement in localization. Fig. 10(f–h) show a dramatic improvement in the source localization accuracy as the DBF maps are now readily interpretable. The nearly circular focal-spot is now centered at the expected source location, although the side-lobe levels are marginally higher than its counterpart maps shown in Fig. 7(f–h), respectively. Furthermore, the focal-spot resolution in Fig. 10(e–h) is given by $3.68\lambda_{0},2.6\lambda_{0},2.42\lambda_{0}$ and $3.2\lambda_{0},$ respectively, which are marginally inferior to the counterpart values for the mixer-grinder DBF maps shown in Fig. 7(e–h), respectively, and is attributed to the semi-reverberant room environment. The CLEAN-SC deconvolution [4] was also implemented on the DBF maps shown in Fig. 10(e–h). Fig. 10(i–l) present the DBF + CLEAN-SC source maps, which show a significant improvement in the source resolution, particularly at lower frequencies while the side-lobes observed at higher frequency in Fig. 10(g) and (h) are now completely suppressed. Consequently, the dominant noise source region can now be more clearly visualized.

The results in parts (e-h) of Fig. 7, Fig. 10 collectively demonstrate the efficiency and the necessity of implementing the CCM windowing-based DBF algorithm for improving the localization accuracy of noise source generated from commonly used engineering applications regardless of the acoustic environment they are placed in. Notably, the results also demonstrate the rule of thumb developed for determining the optimal half-window length: at low frequencies, the optimal half-window comprises only the first few data points, while at higher frequencies, a larger half-window must be considered to ensure that sufficient direct field data is accounted for whilst the reflections are filtered out. Additionally, parts (i-l) of Fig. 7, Fig. 10 further highlight the effectiveness of implementing CLEAN-SC on DBF maps, particularly, in semi-reverberant environments to clearly image the dominant noise source region(s).

### Comparison of the auto-correlation graphs for the mixer-grinder with loudspeaker source

With a view to further highlight the necessity of implementing the DBF algorithm using an optimal window length for localizing noise source(s) from an engineering application placed in a semi-reverberant environment, the auto-correlation graphs of the mixer-grinder and the loudspeaker are compared. To this end, the mixer-grinder was replaced with the same loudspeaker source (SONY SRS-XB12) in the semi-reverberant room, and it was made to emit white noise broadband signals in the frequency range 100 to 7000 Hz. Fig. 11(a) compares the $C_{11}\left(\tau \right)$ graphs for the broadband loudspeaker data with the counterpart result of the mixer-grinder. While it is evident that reflections do occur at regular intervals for the loudspeaker test-case, their magnitudes are indeed very small and may indeed be altogether ignored in comparison to the direct signal peak at τ=0, which explains the small localization error in the CBF maps presented in Fig. 5(d–f). On the other hand, the first few reflection peaks due to the mixer-grinder are prominent and quite comparable to the direct signal peak at τ=0, which explains the complete failure of the CBF in this case. Also, note the large difference in the time-of-occurrence of the first reflection peak for the loudspeaker given by τ≈2.9ms and mixer-grinder given by τ=10.11ms. This suggests that unlike Ref. [15], a simple loudspeaker may not be sufficiently accurate towards determining the optimal frequency-dependent half-window widths for real-world applications, which following the present work has to be determined uniquely using a trial-and-error method.

### Acoustic pressure distribution measured on a near-field plane

**Fig. 12:**
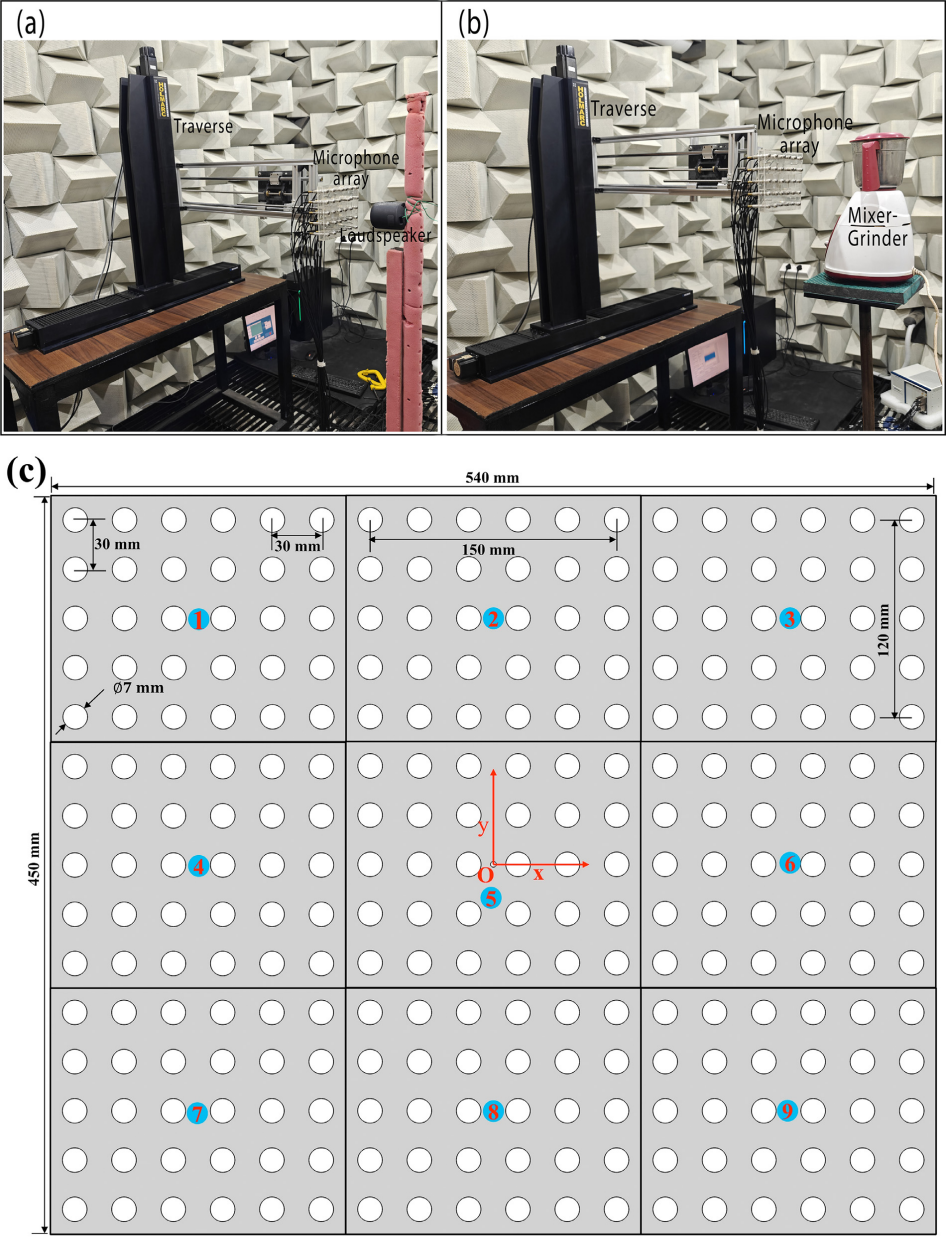
Photographs of the experimental set-up in an anechoic chamber, where a 30-channel microphone array grid was mounted on a traverse to record the near-field acoustic data on a plane at a distance 75 mm from (a) loudspeaker source and (b) a mixer-grinder home-appliance. (c) Schematic depicting the different positions 1 to 9 over which the small rectangular array patch was moved using the traverse to record near-field acoustic data over a plane with much larger dimensions.

Experiments were carried out to measure and plot the acoustic pressure distribution due to the SONY SRS-XB12 loudspeaker and mixer-grinder home appliance on a near-field plane, and the results will be used to corroborate the counterpart DBF+CLEAN-SC source maps shown in Fig. 7(i–l). Fig. 12(a) and (b) show the experimental set-up of the loudspeaker and mixer-grinder in an anechoic chamber, respectively, where a 30-channel microphone array arranged in the form of a small rectangular grid comprising 5 × 6 microphones, i.e., 5 rows and 6 columns of uniformly-spaced microphones were mounted on a high-precision traverse (make: Holmarc Opto-Mechatronic Ltd [26]) having a positional accuracy 20μm to record the near-field acoustic data. The inter-microphone spacing along each row and column was set equal to 30 mm, therefore, the dimensions of the rectangular array patch was 120 mm x 150 mm as annotated in Fig. 12(c). The distance between the array plane and the test-cases was set to 75 mm ensuring that one is in the near-field. Note that logistic constraints prevented measuring the data any closer than this distance. As illustrated in Fig. 12(c), data was recorded sequentially on the rectangular array patch beginning with position 1 (top left), then moving horizontally to record data on positions 2 and 3 (top right). Likewise, data was recorded sequentially at patch positions 4 to 6 (middle row), and finally at patch positions 7 (bottom left) to position 9 (bottom right). As annotated in Fig. 12(c), the overall dimensions of the near-field imaging plane covered by moving the rectangular array patch were −270mm≤x≤270mm and −225mm≤y≤225mm. Note that sequential measurements were possible because the mixer-grinder generates stationary signals when operating at a constant RPM. Furthermore, the origin **O** marked in Fig. 12(c) was chosen to be coincident with the interface between the hub and steel griding jar of the mixer-grinder. The near-field acoustic data of the mixer-grinder was bandpass filtered with half-bandwidth 12.5 Hz centered at 672 Hz, 1008 Hz, 2008 Hz and 4680 Hz, i.e., at roughly the same frequencies as the those considered in Fig. 7(e–h), respectively. The RMS of the bandpass filtered data was computed and plotted on the dB scale on the near-field plane. Similarly, for the loudspeaker, its center was taken as the origin and a white-noise signal was recorded over the near-field array, and the same procedure as the mixer-grinder was followed for processing the near-field acoustic pressure distribution.

**Fig. 13:**
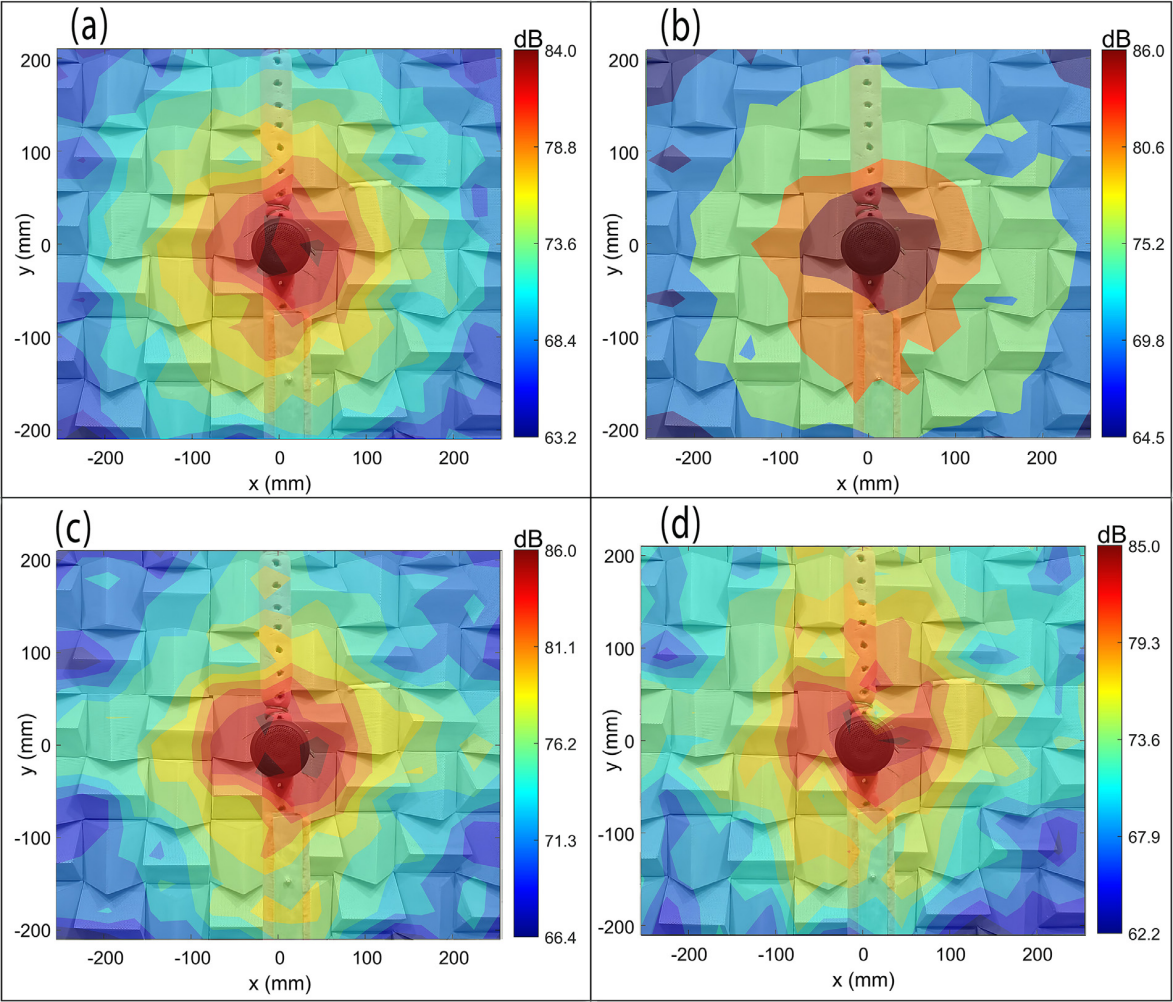
Near-field acoustic pressure distribution measured over a plane at a distance 75 mm from the loudspeaker source at frequency (a) $f_{0}=$ 672 Hz, (b) $f_{0}=$ 1008 Hz, (c) $f_{0}=$ 2008 Hz and (d) $f_{0}=$4680 Hz.

Fig. 13(a–d) show the near-field acoustic pressure distribution due to the loudspeaker at 672 Hz, 1008 Hz, 2008 Hz and 4680 Hz, respectively. It is observed that the local acoustic pressure maxima region is co-incident with the loudspeaker, thereby demonstrating as a proof-of-concept that the near-field acoustic pressure distribution can correctly identify the source location at the loudspeaker – the same conclusion was drawn by analyzing the CBF source maps shown in Fig. 5(a–c) pertaining to the loudspeaker placed in anechoic chamber. However, the difference between CBF and near-field acoustic pressure distribution is that the source resolution is frequency-dependent in the former method and is poor in the low-frequency range while in the latter, one observes that the focal spot size is nearly the same across low-to-high frequencies.

**Fig. 14:**
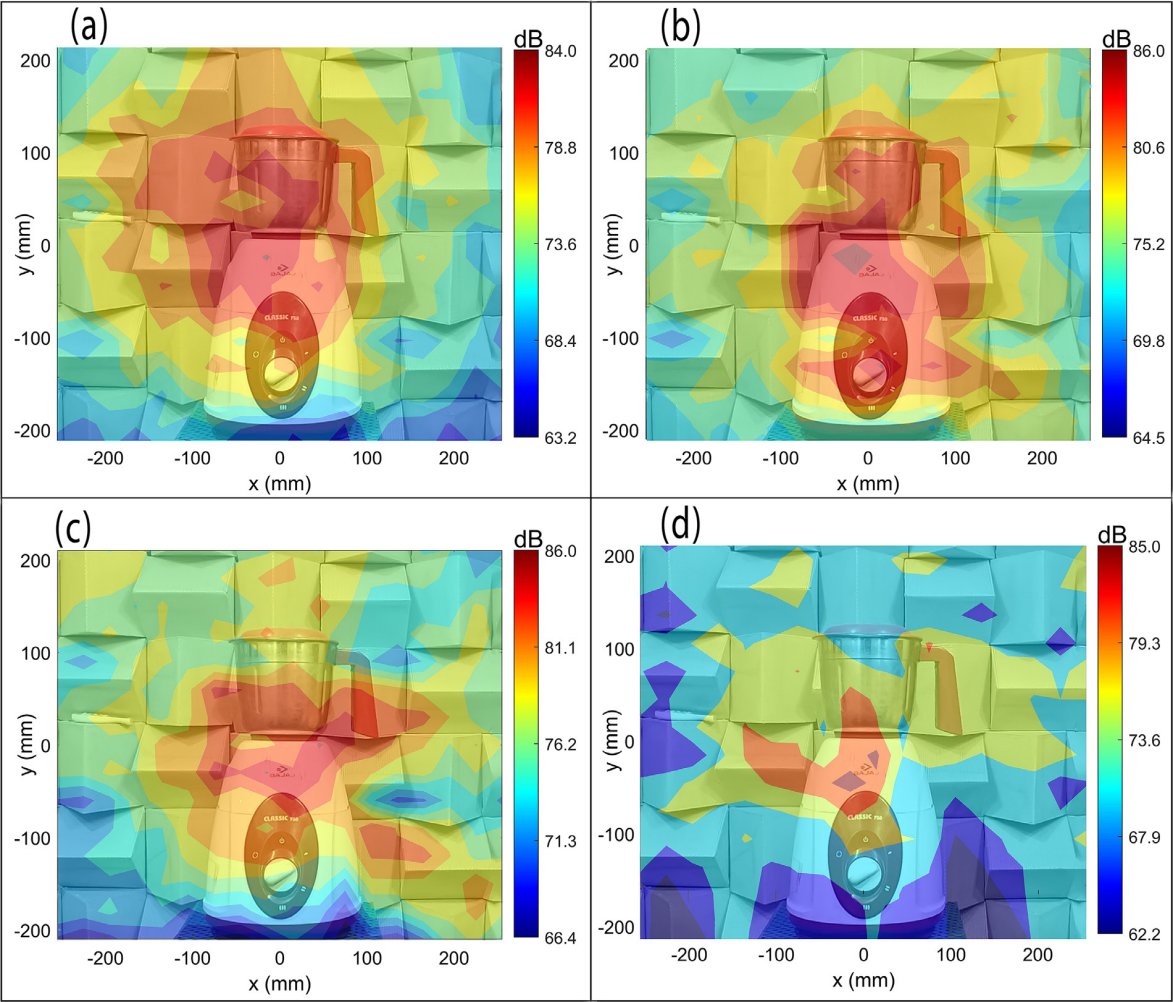
Near-field acoustic pressure distribution measured over a plane at a distance 75 mm from the mixer-grinder home appliance at frequency (a) 672 Hz, (b) 1008 Hz, (c) 2008 Hz and (d) 4680 Hz.

Fig. 14(a–d) present the near-field acoustic pressure distribution plots for the mixer-grinder home appliance at 672 Hz, 1008 Hz, 2008 Hz and 4680 Hz, respectively. It is observed that the acoustic pressure maxima region, i.e., the dominant noise source is localized at the top portions of the white hub that houses the motor and power transmission, and it is observed to be highly comparable with predicted location observed at the top of the hub in Fig. 7(i–l), respectively. Therefore, the acoustic pressure distribution on a near-field imaging plane close to the mixer-grinder surface is consistent with the predicted source location obtained from the counterpart DBF+CLEAN-SC source maps computed on the plane passing through the appliance, thereby corroborating the overall effectiveness of the DBF algorithm across low-to-high frequency regions. In future investigations, one may further validate the DBF+CLEAN-SC source localization maps for an engineering appliance with the near-field acoustic holography (NAH), see Refs. [27,28].

### Concluding remarks

This work has demonstrated the necessity and effectiveness of implementing an improved de-reverberation beamforming (DBF) algorithm based on optimal frequency-dependent cross-correlation matrix (CCM) windowing to improve the noise source localization accuracy in anechoic and semi-reverberant environments. The test-cases of a simple loudspeaker and mixer-grinder home appliance were considered to demonstrate the DBF algorithm. By taking an inverse FFT of the cross-spectral matrix (CSM), the CCM was computed, which allows one to observe the time-of-occurrence of reflection peaks in the τ domain. Next, a Hanning window filter was implemented about the main source peak at τ=0 such that sufficient data pertaining to only the direct field was retained, and the optimal window lengths were determined through a trial-and-error approach for a given frequency, preferably with some prior knowledge of the possible location of the dominant sources.

The optimal truncated window length was shown to significantly vary with frequency – the rule of thumb formulated here suggests that at low frequencies, only the first few data points of the CCM should be considered while an increasingly larger data points should be considered with an increase in frequency. Furthermore, the optimal window length also significantly depends upon the acoustic environment in which the noise source is placed as well as the geometry of the source. For the loudspeaker test-case, the CBF delivered an accurate localization in the anechoic chamber, i.e., windowing was not required while in the semi-reverberant room, a small localization error was observed in the CBF maps, and DBF needs to be implemented to eliminate the error. For the mixer-grinder in an anechoic chamber, this work suggests that the DBF with an optimal half-window must be implemented to remove reflections and self-scattering from the application body recorded by the microphone array - otherwise, a noticeable localization error results in the CBF maps, particularly at higher frequencies. When this application was placed in the semi-reverberant room, the CBF performed even more poorly, rendering it nearly impossible to interpret the source maps due to the occurrence of large side-lobes; however, satisfactory results were obtained when the DBF algorithm was implemented. Furthermore, the optimal half-window widths were found to be substantially different for the loudspeaker and mixer-grinder at comparable frequencies in the semi-reverberant room environment.

The CLEAN-SC deconvolution algorithm [4] was also implemented to improve the resolution of the DBF maps for the mixer-grinder home appliance in both anechoic and semi-reverberant room environments. By suppressing the side-lobes and regions within the focal spot that are coherent with the main lobe, the DBF+CLEAN-SC maps were shown to deliver an enhanced focal spot signifying the dominant noise producing region on the mixer-grinder hub, thereby improving the results, particularly at low-frequencies. The overall effectiveness of the localization accuracy of the DBF+CLEAN-SC algorithms was demonstrated by corroborating with the near-field acoustic pressure distribution, which also showed that the local acoustic pressure maxima was observed on top portion of the hub.

## Limitations

The somewhat tedious and time-consuming nature of determining the optimal half-window length is perhaps, a limitation of the present DBF algorithm because it involves computing and carefully analyzing a sequence of source maps corresponding to truncated half-window of different lengths for a particular frequency band, noise source and a given acoustic environment. In a future investigation, the focus will be on developing customized machine learning algorithms [29] to identify the optimal half-window, which delivers an accurate noise source localization for more complex engineering applications, preferably with some prior knowledge of the most probable source distribution.

## Ethics statements

The methods used in the study did not involve any human or animal for experimentation. Therefore, no ethics statement is applicable in the present work.

## CRediT author statement

**Rohit Singh:** Software, Formal analysis, Investigation, Data curation, Writing - original draft, Visualization. **Akhilesh Mimani:** Conceptualization, Supervision, Formal analysis, Investigation, Funding acquisition, Writing – review & editing. **Randhir Kumar:** Investigation, Formal analysis, Data curation.

## Declaration of competing interest

The authors declare that they have no known competing financial interests or personal relationships that could have appeared to influence the work reported in this paper.

## Data Availability

Data will be made available on request.
